# A remarkable response to pazopanib, despite recurrent liver toxicity, in a patient with a high grade endometrial stromal sarcoma, a case report

**DOI:** 10.1186/s12885-018-3999-0

**Published:** 2018-01-22

**Authors:** Arie J. Verschoor, Fabiënne A. R. M. Warmerdam, Tjalling Bosse, Judith V. M. G. Bovée, Hans Gelderblom

**Affiliations:** 1 Department of Medical Oncology, Leiden University Medical Centre, Albinusdreef 2, Leiden, The Netherlands; 2 Department of Internal Medicine, Zuyderland Medical Centre, Henri Dunantstraat 5, Heerlen, The Netherlands; 3 Department of Pathology, Leiden University Medical Centre, Albinusdreef 2, Leiden, The Netherlands

**Keywords:** Pazopanib, Endometrial stromal sarcoma, Liver function, Adverse events, Response

## Abstract

**Background:**

Pazopanib is an oral tyrosine kinase inhibitor registered for metastatic renal cell carcinoma and soft tissue sarcoma. Liver toxicity is a common side effect for this class of agents. The current opinion is that in case of severe liver toxicity pazopanib should be interrupted and restarted at a lower dose after returning to Common Terminology Criteria for Adverse Events (CTCAE) grade 1. After recurrence of liver toxicity at the lower dose it is advised to permanently stop pazopanib. We describe a patient with an YWHAE-FAM22 translocated endometrial stromal sarcoma with a remarkable response to pazopanib despite recurrent liver toxicity.

**Case Presentation:**

A 40 year old woman was diagnosed with metastatic YWHAE-FAM22 translocated endometrial stromal sarcoma. She was treated successively with doxorubicin, megestrol acetate and anastrozole, before pazopanib was initiated. Several dose interruptions and reductions were necessary due to liver toxicity, but nevertheless she had a good partial response. Seven months after the start, pazopanib was permanently stopped because of a bilateral pneumothorax. Nine months later it was reinitiated because of progression and was continued for another 8 months until final disease progression.

**Conclusion:**

In contrast to the current summary of product characteristics of pazopanib, the drug was successfully continued despite recurrent liver toxicity, and no further liver function deterioration was found. This case suggests that further dose reductions are good practice when liver toxicity limits treatment in responding patients.

Secondly, this patient with rare YWHAE-FAM22 translocated endometrial stromal sarcoma showed a remarkable response to VEGFR/KIT inhibitor pazopanib. Recently, it was reported that this specific subtype of endometrial stromal sarcoma overexpresses CD117, but has no KIT mutations.

This case illustrates that (a) pazopanib can be continued in patients with recurrent liver toxicity after dose reductions under strict surveillance and that (b) pazopanib shows good efficacy in YWHAE-FAM22 translocated endometrial stromal sarcoma.

## Background

The oral anti-angiogenic tyrosine kinase inhibitor (TKI) pazopanib is a multi-target TKI [1]. Its main activity is against vascular endothelial growth factor receptors (VEGFR) 1, 2 and 3, platelet derived growth factor receptors (PDGFR) and KIT [1]. The phase III PALETTE study showed an increase in the progression free survival (PFS) from 1.6 months with placebo to 4.6 months with pazopanib (Votrient®, GlaxoSmithKline) in patients with metastatic soft tissue sarcoma [2, 3]. Pazopanib is also effective in renal-cell carcinoma (RCC), with an increase in PFS from 4.2 months with placebo to 9.2 months with pazopanib [4]. Overall survival (OS) was also significantly increased with pazopanib despite cross-over from placebo treatment to pazopanib treatment [5].

Liver toxicity is a well-known side effect of pazopanib, with an incidence in the PALETTE study of a grade ≥ 2 elevated alanine aminotransferase (ALAT) of 10% (placebo 3%) and aspartate aminotransferase (ASAT) of 8% (placebo 2%) [2]. An increase in total bilirubin was not seen in this study. In the RCC study, a National Cancer Institute (NCI) Common Terminology Criteria for Adverse Events (CTCAE) grade ≥ 3 increase in ALAT was found in 12% (placebo 1%), ASAT 7% (< 1%) and total bilirubin 3% (1%) [4]. Two patients in this RCC study were assessed as having died due to abnormal hepatic function. The current opinion is that physicians should be careful prescribing pazopanib in patients with abnormal ASAT, ALAT and total bilirubin levels and that treatment should be discontinued at least temporarily when grade 3 or more elevations occur during treatment. In case of recurrence of liver function abnormalities after restarting treatment pazopanib should be stopped permanently [6].

Endometrial stromal neoplasms of the uterus are divided into four categories based on the 2014 WHO classification, i.e. endometrial stromal nodule, low-grade endometrial stromal sarcoma (ESS), high grade ESS and undifferentiated uterine sarcoma [7, 8]. ESS is very rare, representing 0.2% of all genital tract malignancies and the patients are generally younger than in other uterine malignancies. Low grade ESS is usually CD10 positive and Cyclin D1 negative [9]. Fifty to 60 % of the ESS harbour translocations involving JAZF1. Recently, it was found that high grade ESS (negative for CD10, positive for Cyclin D1) are characterised by a t(10;17)(q22;p13) translocation resulting in a YWHAE-FAM22 (also known as YWHAE-NUTM2A/B) gene fusion [10, 11]. It is suggested that these tumours have a poor response to therapy compared to the lower grade ESS, which has a 5 year disease specific survival rate for FIGO stage III-IV of 50.3% [12]. Current treatment for metastatic or locally advanced disease consists of hormonal therapy and chemotherapy, i.e. doxorubicin or ifosfamide monotherapy or gemcitabine/docetaxel combination therapy [9]. Pazopanib is also registered for this soft tissue sarcoma subtype, but specific studies are not available and will not be run because of the rarity of this histologic subtype.

This case report describes a patient successfully treated with pazopanib for an ESS developing abnormal liver chemistry, which continued treatment with adapted dosing of pazopanib under careful surveillance of liver function. She had a remarkable response on pazopanib.

## Case Presentation

**Fig. 1:**
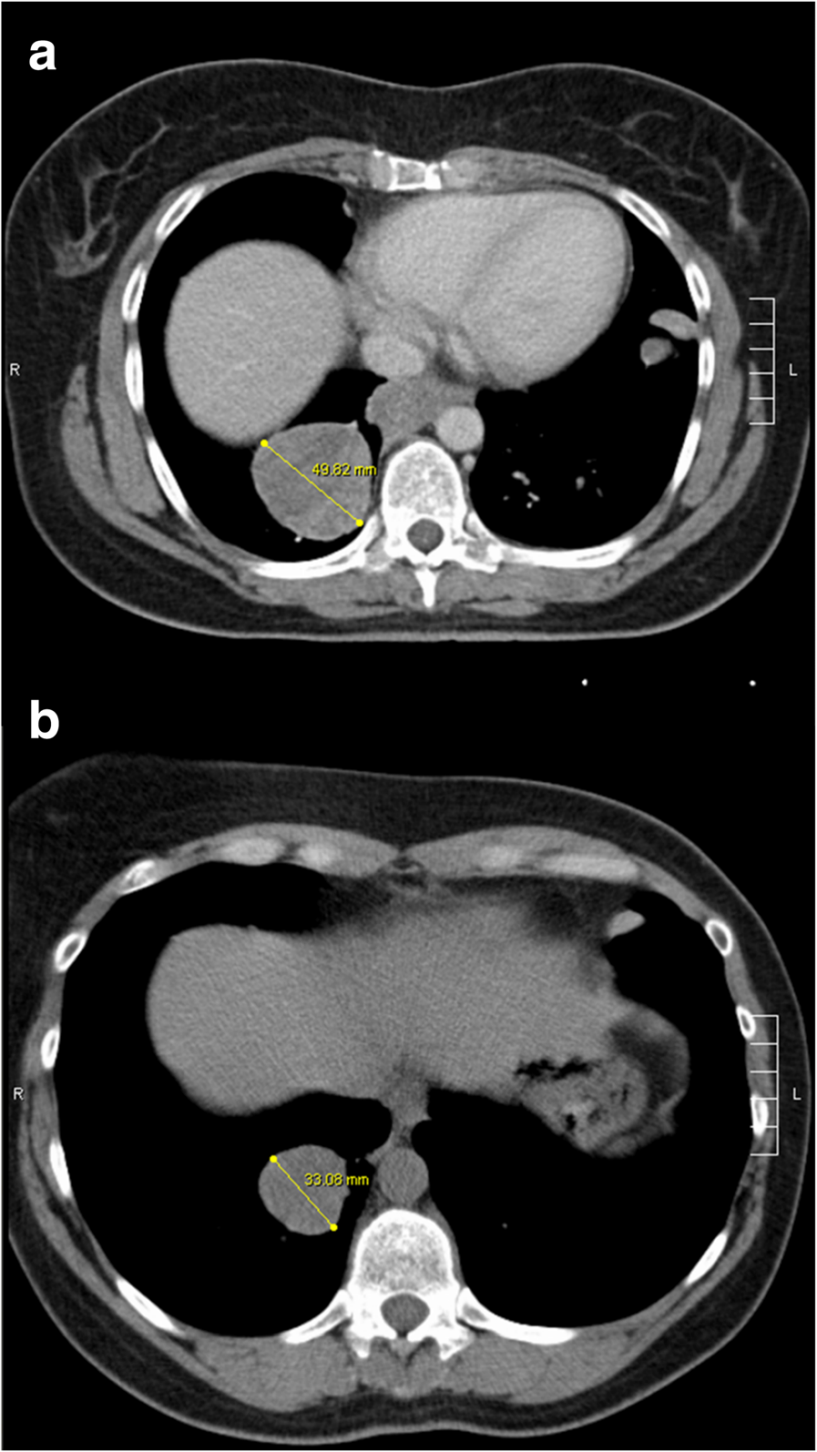
CT scans. Evaluation CT scans before start of pazopanib (**a**) and 12 weeks after start of pazopanib (so 6 weeks on pazopanib and 6 weeks off because of liver toxicity) (**b**) showing a shrinking pulmonary metastasis measured by largest diameter at both times

**Fig. 2:**
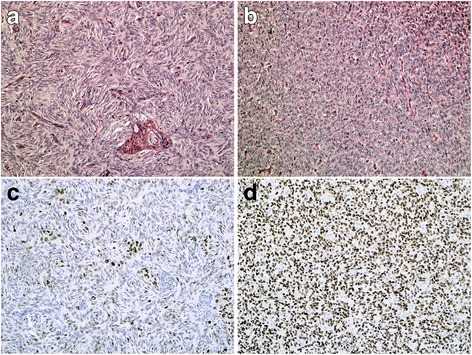
Histology. **a**: Normal HE slides of the lower grade component of ESS, showing spindled cells in a storiform pattern. **b**: HE slides of the high grade component of ESS, showing round cells and open nuclei. **c**: Cyclin D1 expression of the lower grade component is weak and more heterogeneous. **d**: Diffuse and strong Cyclin D1 expression in the high grade areas

A 40 year old female presented with a 4 months history of recurrent right sided lower thoracic pain during sports. Medical history consisted of a hysterectomy for symptomatic uterine myomatosis four years previously, after giving birth to her second child. This was a non-radical non-oncological resection. The original pathology report revealed a 15 cm low grade ESS. Now, four years after the hysterectomy, a CT scan showed a large mediastinal mass with expansion in the upper abdomen and compression of the left atrium and multiple lesions in both lungs. A bronchoscopic biopsy showed cells similar to the earlier diagnosed ESS. Treatment with six courses of doxorubicin resulted in a very good partial response. Maintenance therapy with megestrol acetate 160 mg was started hoping to prolong the time to progression. Two years later, an increase in size and number of the lung metastases was observed, so megestrol acetate was stopped and anastrozole 1 mg started. Further disease progression was noticed 4 months later (Fig. 1a). Pazopanib 800 mg orally once daily was started in the named patient program in collaboration with the Leiden University Medical Centre, one of the Dutch sarcoma reference centres. At the same time, pathology review was performed, confirming the diagnosis of low grade ESS, but also noticing a CyclinD1 positive and CD10 negative higher grade component suggesting a YWHAE-FAM22 translocated ESS. (Fig. 2a-d) Fluorescent in situ hybridisation confirmed the diagnosis. The tumour showed high expression of KIT in the high grade component of the tumour.

**Fig. 3:**
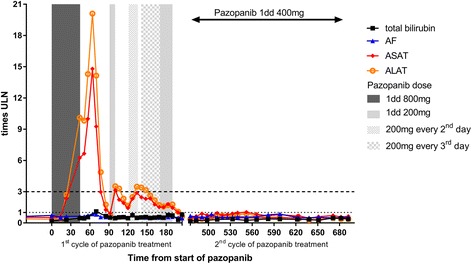
Course of pazopanib dose and laboratory parameters in time. ULN: upper limit of normal

**Fig. 4:**
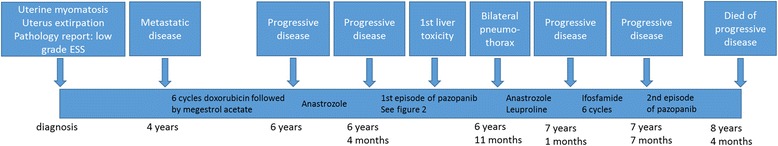
Timeline. Description of treatment in time

Six weeks after start of pazopanib 800 mg once daily an increase in ALAT to > 10 times the upper limit of normal (ULN) was observed. (Figure 3) Hepatitis serology, liver ultrasound and liver biopsy were not performed because of the clear relationship of the increased liver enzymes with the start of pazopanib. She had no history of liver disease, no liver metastases and no liver function abnormalities before start of pazopanib. During the previous treatment with doxorubicin, y-glutamyl transferase increased to 20× ULN and ALAT to 6× ULN, no other liver function abnormalities occurred. The CT scan showed a decrease in the sum of the largest diameters of the target lesions of 23%. Pazopanib was put on hold immediately until the liver enzymes returned to normal. (Figure 3) After six weeks without pazopanib the CT scan showed a further decrease of the target lesions of 38% compared to baseline (19% compared to the previous CT scan) indicating a partial response. Figure 1b) At the time the liver toxicity had almost recovered, a re-challenge with pazopanib 200 mg once daily was performed, but after one week liver function abnormalities recurred and pazopanib was stopped again. After liver function recoverage, pazopanib 200 mg once every other day was initiated, but again after 2 weeks liver enzymes increased to > 3× ULN. Another week later pazopanib 200 mg every third day was given for 2 weeks, until a CT scan showed progression (six months after the start of pazopanib). She was still in very good clinical condition and besides the remarkable inclination of liver enzymes at the beginning of treatment and lesser rises thereafter she had had little other toxicity with pazopanib. She consented to another challenge with pazopanib 200 mg every day under further strict surveillance of liver enzymes. The liver enzymes remained < 3 times ULN, but 3 weeks later she was admitted with a bilateral pneumothorax and pazopanib was stopped. On the right side a pleurodesis was performed and on the left side it was drained. Anastrozole and leuproreline were prescribed, hoping to slow down progression. Two months later, a CT scan again showed progressive disease, and ifosfamide chemotherapy (5 g/m2 in a 24-h continuous infusion every 3 weeks) was initiated. CT scans after 3 and 6 cycles showed stable disease. During ifosfamide treatment only a slight increase in y-glutamyl transferase was noticed (increase to < 2× ULN). Two months after the last ifosfamide administration, radiological progression was observed again and pazopanib 400 mg daily was prescribed again under strict weekly surveillance of the liver enzymes. This dose was chosen because patient had a pleural effusion due to the progression and thereby a lot of complaints. To be sure of a therapeutic dose of pazopanib we started with 400 mg daily. Two weeks after reinitiating pazopanib the pneumothorax on the left side recurred and persisted. Pazopanib was held for two weeks, but was then reinitiated after discussion of the risks with the patient. CT scans 6 weeks and 3.5 months after the restart of pazopanib showed stable disease. Pazopanib was stopped permanently 6 months after the restart because of progressive disease, and she died 2 weeks later. In the second period of pazopanib treatment, no liver toxicity occurred. No differences between co-medication were found between the first period of pazopanib treatment and the second. A summary of the patients’ history is given in Fig. 4.

The DNA of the patient was analyzed for single nucleotide polymorphisms (SNPs) in enzymes known to be involved in the absorption, distribution, metabolism and elimination of pazopanib [13]. The genes analyzed were *CYP3A4, CYP1A2, CYP2C8, ABCB1* and *SLCO1B1.* The main metabolizing enzyme for pazopanib, *CYP3A4* did not have SNPs associated with a decreased metabolic function. Two SNP variants in *CYP1A2* (rs762551 and rs2470890) and one in *SLCO1B1* (rs4149057) were detected, but no evidence exists that these polymorphisms cause elevated levels of pazopanib. So, this analysis did not result in the elucidation of a cause for the liver toxicity in this patient.

## Discussion and conclusion

This case report has 2 new learning messages: we present a patient with an ESS with a remarkable response on pazopanib and we describe clinical management of pazopanib induced recurrent liver toxicity.

Liver function test abnormalities, mainly elevations in ASAT and ALAT, are a common side effect of pazopanib and more in general of TKIs [14]. The exact pathophysiologic mechanism is unknown. A distinctive class effect seems to be unlikely, because pharmacologically different TKIs are known to be hepatotoxic and the substances are also very different chemical compounds. In a case series of two patients with liver toxicity of pazopanib liver histology showed mild active cholestatic hepatitis with inflammation that predominantly involved portal tracts [15]. Recently, it was reported that treatment with pazopanib in combination with prednisolone in case of liver function abnormalities prevented recurrence of liver function abnormalities in two patients [16]. UDP-glucuronosyltransferase isoform 1A1 has been related to bilirubin elevations during pazopanib treatment. These are probably patients with latent Gilbert syndrome becoming evident due to the inhibitory effect of pazopanib [14]. More recently, an association between HLA-B*57:01 and pazopanib induced liver toxicity was found [17]. Whether other germline genetic causes of pazopanib induced liver toxicity exist is unclear. The summary of product characteristics (SmPC) of pazopanib contains guidelines on handling liver toxicity [6]. If the elevation of ASAT and ALAT between 3 and 8× ULN it is safe to continue pazopanib with strict control of ASAT and ALAT until grade 1 toxicity. If ASAT and/or ALAT is more than 8× ULN then pazopanib should be stopped until recovery till grade I or less and if reinitiated restart with pazopanib once daily 400 mg. It should be stopped permanently if ASAT and/or ALAT rise again to > 3× ULN. Pazopanib should also be stopped if ASAT and/or ALAT are elevated > 3× ULN concurrently with a bilirubin elevated > 2× ULN. In our case, despite these recommendations, pazopanib was reintroduced at further reduced dose and no progressive liver insufficiency developed. Ultimately this strategy seemed to be effective until a dose reduction to 200 mg once every second day. Another possible solution for toxicity management would be therapeutic drug monitoring, which is possible for pazopanib with a dried blood spot assay [18]. However, because the intrapatient variability of pazopanib levels is high, a recent study could not show an effect of therapeutic drug monitoring on the interpatient variation [19]. A SNP analysis of genes for enzymes known to be involved in the metabolism of pazopanib did not provide an explanation for elevated pazopanib levels or liver toxicity in our patient. In case of further dose reductions after reduction to once daily 400 mg, we suggest that patients should be monitored closely with once weekly liver function testing.

The second message of this case report regards the pazopanib response related to the tumour type. When feasible ESS is treated with radical surgery. Treatment of metastatic or locally progressive ESS consists of endocrine therapy as first line and when progressive on endocrine therapy chemotherapy. No specific data on the use of pazopanib in ESS is available, but this case report shows that pazopanib induced a partial response and prolonged PFS of 9 months which is more than the median PFS in the PALETTE study. Although in general ESS does not overexpress KIT, it was recently shown that ESS harbouring the YWHAE-FAM22 fusion gene frequently overexpress KIT (without a mutation in the KIT oncogene) in the high grade component of the tumour, as was the case in our patient [11, 20]. This could explain the good response, in this rare histologic subtype, to pazopanib, which is an inhibitor of VEGFRs, PDGFRs and KIT. Based on these findings there may also be a potential role for imatinib, which was already reported in two cases [21, 22]. Most probably the evidence for treatments in ESS will be not better than small case series due to the rarity of this disease.

The 3rd teaching point of this case report regarding the occurrence and clinical development of a pneumothorax during pazopanib treatment in (responding) patients with pleurally located metastatic lesions was addressed in a previous case series [23].

In conclusion this case illustrates two important new points:

- First, reversible severe liver toxicity with pazopanib treatment is a known rare side effect of pazopanib and low dose personalized rechallenge in responding patients is a therapeutic option in experienced hands. This low dose personalized rechallenge is in conflict with the current SmPC of pazopanib and patients should be closely monitored for liver function abnormalities with for example weekly testing of the liver functions.
- Second, pazopanib was found to result in a good response in this patient with a YWHAE-FAM22A/B translocated ESS which may be related to the KIT overexpression in these tumours.

## Abbreviations

ALAT: Alanine aminotransferase
ASAT: Aspartate aminotransferase
CTCAE: Common Terminology Criteria for Adverse Events
ESS: Endometrial stromal sarcoma
NCI: National Cancer Institute
OS: Overall survival
PDGFR: Platelet derived growth factor receptors
PFS: Progression free survival
RCC: Renal-cell carcinoma
SmPC: Summary of product characteristics
SNP: Single nucleotide polymorphism
TKI: Tyrosine kinase inhibitor
VEGFR: Vascular endothelial growth factor receptors
